# Comprehensive, multisystem, mechanical decolonization of Vancomycin-Resistant Enterococcus and Carbapenem-Resistant Enterobacteriacease without the use of antibiotics

**DOI:** 10.1097/MD.0000000000023686

**Published:** 2021-01-22

**Authors:** Eunseok Choi, Sook Joung Lee, Sangjee Lee, Jinseok Yi, Yeon Soo Lee, So-youn Chang, Ho Young Jeong, Yunwoo Joo

**Affiliations:** a Department of Physical Medicine and Rehabilitation; b Department of Neurosurgery; c Department of Radiology, College of Medicine, The Catholic University of Korea, Seoul, Republic of Korea, Seoul, Republic of Korea.

**Keywords:** carbapenem-resistant Enterobacteriaceae, decolonization, mechanical evacuation, multidrug-resistant organisms, vancomycin-resistant enterococci

## Abstract

Among multidrug-resistant organisms (MDROs), Vancomycin-resistant Enterococcus (VRE), and Carbapenem-resistant Enterobacteriaceae (CRE) have become major nosocomial pathogens that are endemic worldwide. If VRE/CRE are present as colonizing organisms but do not act as pathogens, these organisms do not cause symptoms and do not require antibiotic use. However, once gastrointestinal colonization with VRE/CRE occurs, it can persist for long periods and serve as a reservoir for transmission to other patients. Therefore, a breakthrough strategy to control the spread of MDRO colonization is needed. We herein introduce decolonization method, which is a comprehensive, multisystem, consecutive mechanical MDRO decolonization protocol that does not utilize antibiotics.

Our protocol included

(1) Mechanical evacuation using a glycerin enema,
(2) Replacement of the normal gut flora using daily lactobacillus ingestion,
(3) Skin hygiene cleansing using chlorhexidine, and
(4) Environmental cleansing by changing the bed sheets and clothing every day.

These steps were repeated consecutively until the patient was released from quarantine. We conducted VRE/CRE tests every week. Because our protocol was a comprehensive and multisystem decolonization protocol, the cooperation of patients and/or caregivers was essential, and family support was important for patient care. Patients were divided into VRE and CRE groups and were subdivided into success and failure groups according to decolonization status.

Thirty-two patients with VRE or CRE colonization were enrolled, and our protocol was performed. A total of 20 patients (62.5%) were successfully decolonized after repeated protocols. Univariate analysis revealed that patients with younger age, higher body mass index (BMI), shorter period of MDRO isolation without trial, and higher functional status showed significantly enhanced success rates with our decolonization protocol.

This study presents the decolonization effects of a comprehensive, multisystem, mechanical decolonization protocol for VRE and CRE. Most importantly, our decolonization protocol does not use antibiotics and is thus not harmful. These results suggest an active decolonization trial to be performed as early as possible in patients with VRE or CRE colonization. This simple, easy-to-apply protocol can be used as 1 of the basic treatment options for MDROs infection or colonization, regardless of whether it requires antibiotic treatment.

## 1 Introduction

In late 2019, the world experienced pandemic outbreaks of COVID-19. We learned the importance of hand washing and hygiene and social distancing.^[1]^ The global emergence and spread of multidrug-resistant organisms (MDROs) are also of great concern to public health. Among the MDROs, Vancomycin-resistant Enterococcus (VRE) and Carbapenem-resistant Enterobacteriaceae (CRE) have become major nosocomial pathogens that are endemic worldwide. VRE has become a major nosocomial pathogen that is endemic worldwide since its first detection in England and France in 1986.^[2–4]^ CRE has also been reported worldwide since it was first identified in 1993 in the USA.^[5,6]^ The spread of CRE is 1 of the most recent and major threats and has become a serious healthcare issue in many countries.^[7]^

If VRE or CRE are present as colonizing organisms but do not acting as a pathogen, these organisms do not cause symptoms and do not require antibiotic use. However, once gastrointestinal (GI) colonization with these organisms occurs, it can persist for long periods and serve as a reservoir for transmission to other patients.^[4,5,8]^ Natural clearance of MDRO colonization takes a very long time; in the case of VRE or CRE, it takes from 204 to 1371 days according to previous studies.^[9–12]^ Prolonged asymptomatic colonization of VRE and CRE in the GI tract and lack of an adequate decolonization regimen perpetuate the endemicity of MDROs.^[2,13,14]^

Most previous VRE and CRE decolonization trials have used antibiotics However, an increase in antibiotic use will cause an increase in antibiotic resistance, which leads to a greater risk of therapeutic failure and worse clinical outcomes. Precautions should also be taken against the considerable “rebound effect” of select antibiotic-resistant organisms in the lower GI tract after discontinuation of the decolonization protocol.^[15]^ Currently, decolonization using antibiotics is not recommended.^[7]^

Therefore, controlling the spread of MDRO colonization and preventing colonized patients from becoming infected are critical issues. In many countries, at the national level, the Centers for Disease and Control and Prevention have developed infection control measures that aim to prevent MDRO transmission.^[14,16,17]^ Although the transmission of MDROs is frequently recognized in many countries, there are no effective decolonization regimens for nosocomial colonization by these bacteria.^[2,3,18]^. The only reported Public Clinical Guidelines for nosocomial infection by these MDROs are

(1) isolation of the patient,
(2) hand hygiene,
(3) contact precautions using gloves and aprons, and
(4) cleaning of equipment and the use of dedicated patient equipment, such as stethoscopes.^[17,19,20]^

However, these guidelines could not directly affect the decolonization of MDROs. Therefore, an effective protocol for VRE and CRE decolonization is fundamental in healthcare settings.^[14,19]^

We introduce a comprehensive, multisystem, serial mechanical decolonization method for VRE and CRE. Our strategy, which is distinct from other studies, carried out repeated mechanical enemas. Most importantly, we do not use antibiotics. We hypothesized that the most common colonization site of both VRE and CRE is the lower GI tract; thus, our strategies for the mechanical evacuation of feces would be effective for both VRE and CRE.

## 2 Methods

### 2.1 Subjects

This study was designed as a preliminary, prospective study. Among the patients who were isolated due to colonization of VRE and CRE organisms, those who were transferred to the Department of Rehabilitation Medicine were recruited in this study. Because our protocol was a comprehensive and multisystem decolonization protocol (from fecal evacuation to environmental clearing), the cooperation of patients and/or caregivers was essential, and family support was important for patient care. Thus, the patients who were in poor condition or were uncooperative could not perform our protocol. Enrolled patients had consistent caregivers who understood and participated in our decolonization protocol.

During the study period, our hospital had over 100 patients who were diagnosed with VRE or CRE, some of which acted as pathogens, and these patients needed to use antibiotics. On the other hand, some patients who could ambulate and had good function with only VRE or CRE colonization were discharged home.

The study protocol was approved by the institutional review board of our institute and all participants provided written informed consent (The Catholic University of Korea, No. DC20RASI0016). The patients provided written and informed consent to allow the publication of their case details.

### 2.2 Definitions of Colonization and Decolonization of VRE or CRE organisms

VRE and CRE colonization and decolonization were defined according to the Korea Centers for Disease Control and Prevention guidance^[17]^ and based on the Clinical and Laboratory Standards Institute (CLSI).^[21]^ Colonization means the presence, growth and multiplication of microorganisms without observable signs or symptoms of infection.^[22]^

VRE is indicated when the minimum inhibitory concentration of vancomycin is greater than or equal to 32 μg/mL. It is also defined by a diameter of less than or equal to 14 mm with the disk diffusion method on a Mueller-Hinton agar plate, according to the CLSI (M100-S27). CRE is defined as Enterobacteriaceae organisms having a low susceptibility to carbapenem. The CLSI standard (M100-S027) used to determine CRE is a minimum inhibitory concentration of > 4 μg/mL for imipenem and meropenem or > 2 μg/mL for ertapenem. CRE is also indicated by a diameter of less than or equal to 19 mm with the disk diffusion method on a Mueller-Hinton agar plate. VRE or CRE carriage was identified through the weekly active surveillance of rectal cultures. If needed, blood, stool, urine, or wound discharge samples were gathered for screening and diagnosis. We did not classify VRE and CRE according to the microorganism. All VRE strains were found to be *Enterococcus faecium*, according to agar medium tests. CRE strains were detected as *Klebsiella pneumoniae* (6 patients), *Proteus mirabilis* (2 patients), *Escherichia coli* (1 patient), and *Enterobacter cloacae* (1 patient).

In Korea, MDRO decolonization is defined as negative findings detected sequentially 3 times every week for 3 weeks for both VRE and CRE.^[19,23]^ Then, the patient is released from quarantine.

### 2.3 Comprehensive, Multisystem Decolonization protocol (M. R. S. E.)

Attending physiatrists explained our decolonization protocol to the patients and their caregivers. Our comprehensive, multisystem, consecutive mechanical decolonization method included

**Figure 1:**
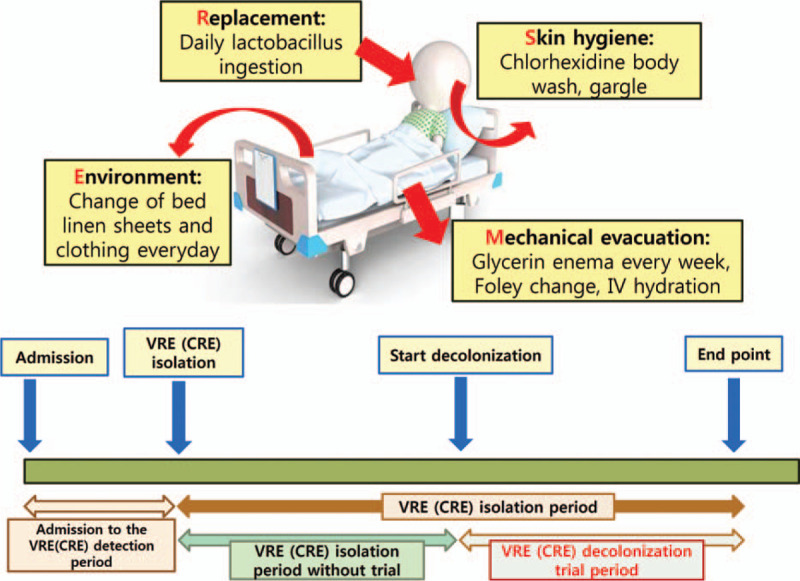
Illustration of comprehensive, multisystem, mechanical decolonization protocol.

(1) Mechanical evacuation using glycerin enemas,
(2) Replacement of the normal gut flora and nutrition support,
(3) Skin hygiene cleansing, and
(4) Environmental cleansing, referred to as the M.R.S.E. protocol (Fig. 1).

This protocol was conducted repeatedly until patients were released from the quarantine.

Mechanical evacuation was performed to artificially wash out GI feces. The patients received a glycerin enema every week, 1 day before rectal swabbing for VRE and CRE examination. Intravenous (IV) hydration using normal saline was administered 1 day before urine culture. If the patient had an indwelling Foley catheter, it was also changed every week.

For replacement of the normal gut flora, patients were administered a Medilac-DS (Hanmi, Seoul, Korea) enteric coated capsule 3 times per day. The 250 mg Medilac-DS capsule included *Bacillus subtilis* and *Enterococcus faecium*. Most patients were sarcopenic status, and additional protein was administered for nutritional support.

Skin cleansing was performed by daily bathing using Microshield 2 Chlorhexidine Skin Cleanser (BL&H, Seoul, Korea) and a Hexamedine solution (Bukwang, Seoul, Korea) gargle that contains chlorhexidine gluconate.

Environmental cleansing was performed daily by changing the patient's clothing, linen, and bed sheets. It also included disinfection of the patient's surrounding environment, such as the bed, suction bottle, commode, and medical devices (with which the patients and their caregivers often made contact), using disinfectants. The isolation room was thoroughly sterilized using 500 ppm Biospot (Neochemical, Seoul, Korea) every day according to the policy of the Department of Infection Control in our hospital.

### 2.4 Evaluation

Patients with VRE and CRE colonization underwent rectal cultures every week. Fecal specimens were cultured on agar medium. These organisms can also be found in the urinary tract associated with urethral catheters, on skin surfaces such as the face or lower half of the patient's body, and even in wounds. Thus, if VRE and CRE were identified from blood, stool, urine, or wound discharge samples, they were also cultured.

Each patient functional status was evaluated using the Korean version of the modified Barthel index, functional ambulatory category (FAC), grip strength power, mini-mental state exam scores, and body mass index (BMI).

Swallowing function was evaluated via a video fluoroscopic swallowing study. Based on the results of the video fluoroscopic swallowing study, patients’ feeding methods were decided as follows: nonoral feeding, limited diet, or normal regular diet. Voiding function was categorized as Foley catheterization, use of diapers without a Foley catheter, and voiding in a toilet.

To distinguish the mechanical decolonization period, we divided the periods as follows: the period from admission to VRE/CRE detection and the period of VRE/CRE isolation. The isolation period was also divided into VRE/CRE isolation periods without protocol trials and periods of decolonization protocol trials (Fig. 1).

Patients were divided into successful and failed decolonization groups according to their final decolonization status. Patients were subdivided into success and failure groups according to the colonizing organisms, namely, VRE and CRE. If patients were transferred to another hospital or were still colonized until 6 months after the protocol, these patients were assigned to the failed group.

Many complaints occurred among the caregivers and nurses due to the daily room cleansing and changes in linen and patient clothing. However, hygienic care and environmental clearing are essential for this decolonization protocol, and these complaints would not be classified as side effects.

### 2.5 Statistical analysis

SPSS 24.0 (IBM Corp., Armonk, NY) was used for statistical analysis. Continuous data are expressed as the mean ± standard deviation (SD), and categorical data are expressed as numbers.

The paired *t*-test or the Mann-Whitney *U* test were used to compare clinical characteristics and related factors between the successful and failed decolonization groups. Fisher exact test was used for categorical variables. The effect of independence of clinical characteristics and functional status on successful decolonization was analyzed using the univariate and multivariate logistic regression analysis. A *P*-value less than .05 was considered statistically significant.

## 3 Results

### 3.1 Overall outcome of the declolonization protocol

**Figure 2:**
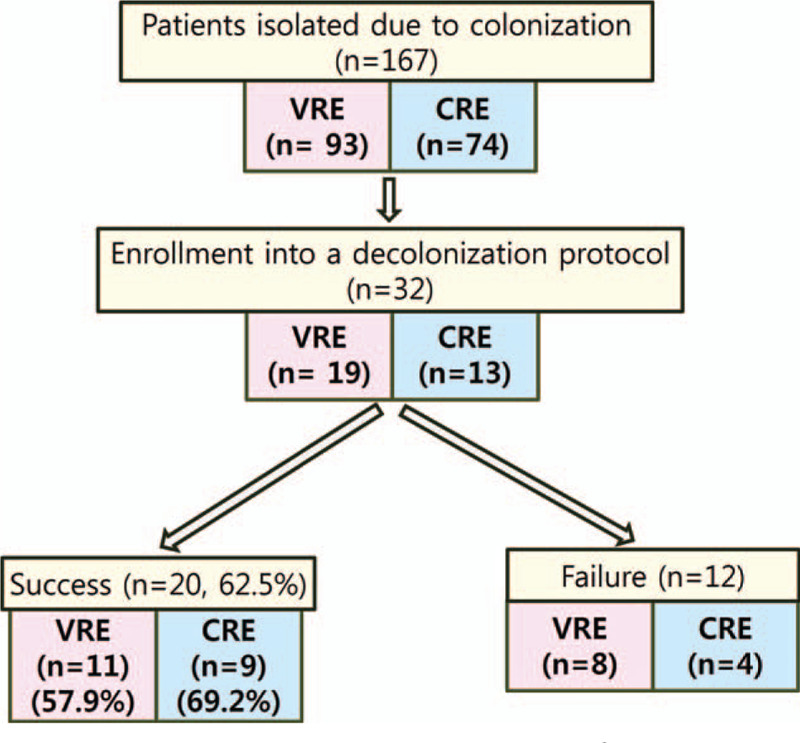
A flowchart of the patients with VRE and CRE colonization.

There were 167 patients who were detected to have VRE and CRE at our hospital during the study period. Finally, thirty-two patients with VRE or CRE colonization were enrolled, and our protocol was performed. Among them, 19 patients with VRE and 13 patients with CRE were enrolled in our decolonization protocol. After the decolonization trial, a total of 20 patients (62.5%), 11 (57.9%) of the 19 VRE colonization patients and 9 (69.2%) of the 13 CRE colonization patients, were successfully decolonized (Fig. 2). In the failed decolonization group, most patients dropped out during the protocol because they received antibiotic therapy related to aspiration pneumonia, ischemic colitis etc., and some patients were transferred to another hospital. These patients could not continue our decolonization protocol; thus, they were classified into the failed group. One VRE carriage patient with esophageal cancer expired due to aspiration pneumonia during the protocol; however, the cause of death was not related to our protocol. He was classified into the failed VRE decolonization group.

### 3.2 Demographic characteristics and factors related to release from quarantine

Table 1 shows demographic characteristics and factors related to release from quarantine. In Table 2, these variables were subdivided according to the VRE and CRE organisms. Patients in the group that was successfully decolonized by our protocol had a significantly younger age, higher BMI score, shorter MDRO isolation period without trial, and higher functional status than patients in the failed decolonization group (Table 1).

**Table 1:** Demographic characteristics and relating factors to release from quarantine.

	Success in decolonisation (n = 20)	Fail to decolonisation (n = 12)	*P*-value
Age^∗^	67.24 ± 12.30	78.56 ± 3.47	.032
Sex (male/female)	12/8	5/7	.679
BMI^∗^	19.61 ± 4.24	16.72 ± 2.53	.016
Bacteria (VRE/CRE)	11/9	8/4	
Detected organ (stool/urine/wound)	15/5/0	8/3/1	.258
Admission to VRE /CRE detect (weeks)	13.33 ± 12.41	16.89 ± 15.75	.465
VRE/CRE isolation period (weeks)	15.00 ± 11.03	25.56 ± 10.94	.047
VRE/CRE isolation period without protocol trial. (weeks)^∗^	5.60 ± 4.84	13.56 ± 8.13	.005
Period of decolonisation protocol trial (weeks)	9.40 ± 7.07	12.44 ± 7.23	.248
K-MBI^∗^	56.53 ± 25.28	27.44 ± 12.82	.003
FAC^∗^	2.80 ± 1.37	1.11 ± 0.60	.003
Grip power^∗^	17.59 ± 7.18	11.28 ± 5.92	.009
Feeding method (non-oral/LD/RD)	2/7/6	4/4/1	.168
Bladder function (Foley/diapers/toileting)	2/8/5	7/2/0	.008

**Table 2:** Demographic characteristics and relating factors according to the organisms.

	VRE		*P*-value	CRE		*P*-value
Decolonization Organism	Success in VRE (n = 11)	Fail in VRE (n = 8)		Success in CRE (n = 9)	Fail in CRE (n = 4)	
Age^∗^	67.75 ± 12.30	79.17 ± 3.97	.037	62.71 ± 19.09	77.33 ± 2.31	.022
Sex (male/female)	5/3	3/3	1.000	2/5	0/3	.467
BMI	20.28 ± 3.47	16.78 ± 0.50	.061	18.70 ± 1.47	16.96 ± 1.10	.171
Disease	Stroke (n = 6)	Stroke (n = 4)		Stroke (n = 5)	Stroke (n = 2)	
	Parkinsonism (n = 1)	TBI (n = 1)		Brain abscess (n = 2)	Parkinson (n = 1)	
	TBI (n = 1)	Esophageal cancer (n = 1)		AKA (n = 1)	Hip fracture (n = 1)	
	Hypoxic brain damage (n = 1)	TB spondylitis (n = 1)		C-myelopathy (n = 1)		
	Cardiac op (n = 1)	Compression fracture (n = 1)				
	Esophageal rupture (n = 1)					
Detected organ (stool/urine/wound)	8/3/0	6/2/0	.361	6/3/0	1/2/1	.571
Admission to VRE /CRE detect (weeks)	13.50 ± 16.03	18.77 ± 19.25	.435	13.14 ± 7.73	13.33 ± 6.11	.908
VRE/CRE isolation period (weeks)	17.43 ± 14.61	26.82 ± 10.76	.174	12.57 ± 4.65	19.40 ± 9.64	.302
VRE/CRE isolation period without protocol trial. (weeks)^∗^	6.13 ± 6.23	16.93 ± 8.01	.024	4.43 ± 2.51	8.40 ± 2.65	.007
Period of decolonization protocol trial (weeks)	10.50 ± 8.85	12.33 ± 6.09	.560	8.14 ± 4.67	12.67 ± 10.79	.643
K-MBI^∗^	61.25 ± 25.07	24.07 ± 12.39	.008	51.14 ± 26.35	34.33 ± 12.90	.030
FAC^∗^	3.38 ± 1.30	1.33 ± 0.52	.006	2.14 ± 1.21	0.67 ± 0.58	.007
Grip power^∗^	19.91 ± 8.17	9.97 ± 3.75	.014	18.80 ± 9.65	12.47 ± 1.65	.041
Feeding method (non-oral/LD/RD)	2/4/2	3/2/1	.801	0/3/4	1/2/0	.250
Bladder function (Foley/diapers/toileting)	2/3/3	4/2/0	.199	0/5/2	3/0/0	.008

### 3.3 Univariate logistic regression analysis

Univariate logistic regression analysis revealed that patients with younger age, higher BMI score, shorter MDRO isolation period without trial, and higher functional status showed significantly increased success rates in our decolonization protocol (Table 3). Most importantly, the FAC score was the most powerful success factor of the decolonization protocol among multiple functional factors, and it also significantly affected the multivariate logistic regression analysis.

**Table 3:** Univariate analysis logistic regression relating factors to release from quarantine.

		95% CI		
Variables	Odds Ratio	Lower	Upper	*P*-value
Age	0.834	0.728	1.004	.035
BMI	3.843	0.945	15.630	.040
Admission to VRE /CRE detect (wk)	0.981	0.922	1.043	.532
VRE/CRE isolation period (weeks)	0.920	0.847	0.999	.037
VRE/CRE isolation period without protocol trial. (weeks)^∗^	0.820	0.685	0.981	.03
Period of decolonization protocol trial (wk)	0.940	0.834	1.060	.310
K-MBI^∗^	1.081	1.011	1.156	.023
FAC^∗^	6.706	1.256	35.804	.009
Grip power^∗^	1.341	1.034	1.740	.027

We evaluated the effect of our protocol according to the VRE and CRE organisms. The difference in the effectiveness of our decolonization methods between VRE and CRE was noticed in several variables (Tables 2 and 4). However, the number of failed decolonizations in the CRE group was extremely small, only 4; thus, the result should be interpreted cautiously.

**Table 4:** Univariate analysis logistic regression relating factors to release from quarantine according to organism.

		95% CI				95% CI		
Variables	Odds Ratio	Lower	Upper	*P*-value	Odds Ratio	Lower	Upper	*P*-value
Age	0.862	0.724	1.027	.066	0.839	0.580	1.212	.350
BMI	2.892	0.425	19.685	.078	4.481	0.535	37.526	.167
Admission to VRE /CRE detect (wk)	0.981	0.918	1.048	.566	0.996	0.812	1.220	.967
VRE/CRE isolation period (wk)	0.931	0.849	1.021	.130	0.842	0.641	1.105	.214
VRE/CRE isolation period without protocol trial. (wk)^∗^	0.820	0.671	1.002	.042	0.652	0.345	1.231	.187
Period of decolonization protocol trial (wk)	0.966	0.835	1.118	.644	0.901	0.729	1.114	.336
K-MBI^∗^	1.113	1.001	1.237	.041	1.044	0.960	1.135	.318
FAC^∗^	7.021	0. 146	40.753	.038	>999.999	<0.001	>999.999	.906
Grip power^∗^	1.477	1.005	2.169	.037	1.182	0.855	1.633	.311

Multivariate-analysis logistic regression was performed for variables with a *P*-value < .05 on univariate analysis, which were entered into the model selection procedure using a backward stepwise process. According to the multivariate analysis, FAC and modified Barthel index were independent factors of successful decolonization regardless of the organism.

### 3.4 Side effects

There were few side effects during our protocol. One male patient with atypical Parkinsonism (H & Y stage 5, and FAC level 2) received intensive care unit (ICU) care immediately after a glycerin enema. The patient had received a weekly enema without complications during the initial trial period. After 3 to 4 months of the trial, he showed reduced blood pressure due to the vasovagal reflex immediately after the glycerin enema 3 consecutive times and received ICU management. During his ICU stay, the patient did not receive antibiotic therapy. After adjusting the Parkinson drug, his blood pressure did not decrease any more after the enema procedure. Finally, he was successfully decolonized 5 months after implementing the multisystem mechanical decolonization protocol. One of the female patients with a brain abscess (FAC level 5) complained about tooth stains caused by chlorohexidine gargles. She was also successfully decolonized.

## 4 Discussion

4.1 Comprehensive, Multisystem, Decolonization protocol

This preliminary study revealed the effects of a comprehensive, multisystem, consecutive mechanical decolonization protocol for VRE and CRE colonization. Most importantly, our decolonization protocol does not use antibiotics, which fundamentally distinguishes it from other decolonization studies.^[2,14,22,24]^ Previous studies using antibiotics for decolonization showed variable results.^[2,24,25]^ Overall observed rate of MDROs clearance was found to be 33% to 100% in patients treated with antibiotics. In contrast, through our protocol, 62.5% were successfully decolonized without using antibiotics. Although our protocol modified and combined previous successful decolonization methods, this trial is the first study to evaluate a comprehensive, multisystem, consecutive mechanical decolonization protocol. Our M. R. S. E. protocol includes entire domains, from fecal evacuation to environmental cleansing. This protocol was conducted repeatedly until patients were released from the quarantine, thus a multidisciplinary effort was needed.

This protocol takes time for successive decolonization; however, it is easy to apply in the clinical setting and does not harm patients. Furthermore, to the best of our knowledge, few studies have evaluated the success rate of decolonization protocols associated with functional status. Patients with younger age, higher BMI score, and higher functional status showed significantly improved success rates in our decolonization protocol. Finally, our results also revealed that a shorter MDRO isolation period without a trial led to significantly enhanced and successful decolonization.

### 4.1 Artificial Mechanical evacuation and Replacement of normal gut flora

The most important part of our protocol is mechanical evacuation of colonized VRE and CRE organisms. Understanding VRE and CRE is key for decolonization. The major reservoir of VRE and CRE is the lower GI tract,^[19]^ as the term ‘enteron’ originates from Greek, which means ‘the gut or intestine^[8]^; consequently, these organisms are commonly found in the feces of colonized patients. Thus, we performed artificially lower GI evacuation using glycerin enemas weekly. Cheng study conducted bowel preparation only once, but with a high success rate. Their protocol included antibiotic use and even a room change. However, most of our studied patients had severe disabilities, such as brain disorders and swallowing difficulties; thus, they could not ingest 2 to 4 L of bowel preparation fluid. Therefore, we modified Cheng protocol and administered a weekly glycerin enema to mechanically evacuate the lower GI tract. Washing out feces via repeated enemas seemed to reduce the concentration of these colonized organisms. Indeed, in the cultures from rectal swabs, the concentration of VRE was gradually decreased and finally not detected after repeated use of our protocol. The results of this study objectively indicated that the amount of VRE/CRE colonization was quantitatively decreased by the repetitive mechanical washing step.

To provide supplementation after mechanical evacuation, we replaced the normal gut flora using probiotic enteric-coated capsules containing *Bacillus subtilis* and *Enterococcus faecium*, as adjunctive therapy. Probiotics are live microorganisms that provide health benefits when ingested, generally by improving or restoring the natural balance of the bacteria in the gut flora.^[26]^ Many previous studies have demonstrated the effect of probiotic therapy on the decolonization regimen of MDROs.^[2,7,27]^ Suggested mechanisms of probiotics include maintaining the microbial gut microflora and protecting against pathogens through antimicrobial activity. Zhong et al found that 2 strains of Medilac-S (probiotics) specifically decreased and prevented the growth of various enteric bacterial pathogens.^[28]^ Alternatively, the consumption of monosaccharides could lead to slowed growth of MDROs and a lowered colonic pH.^[27]^ It also improves immune system function.^[29]^

Moreover, most enrolled patients were in a sarcopenic state with malnutrition; thus, additional protein was administered for nutritional support. Most enrolled patients’ BMIs were below the normal ranges, and regression analysis also revealed that patients with lower BMIs failed to significantly respond to the decolonization protocol.

### 4.2 Isolation with Skin hygiene and Environmental cleansing.

A core component of MDRO control strategies are contact precautions and isolation of colonized patients to prevent further contamination.^[17,19,20]^ Transmission of these VRE and CRE strains in the hospital setting can occur via contact with health care workers and the environment.^[30]^ These organisms may contaminate the environment around a patient and can survive in the environment for several days to weeks.^[22]^ Thus, our protocol included skin hygiene and environmental cleansing with isolation. Enrolled patients were sanitized using a chlorhexidine body wash and gargle for skin and oral hygiene, and daily room cleansing was performed using Biospot and linen changes. Several recent publications have shown that the incidence of MDRO acquisition among patients in critical care units can be reduced by the daily use of chlorhexidine-impregnated body washes and gargles.^[4,22,31]^ The isolation room was thoroughly sterilized using 500 ppm Biospot every day, which exerts broad-spectrum and sterilizing activities against MDROs and has no smell. In the COVID-19 outbreak, hand washing and social distancing have been shown to be very crucial to prevent spread.^[1]^ Our decolonization protocol, which includes skin hygiene and environmental isolation with cleansing, is consistent with the preventive strategies against COVID-19.

### 4.3 The Disadvantages and Burden of MDROs

The disadvantages and burden of MDROs are increasing in all fields and affect patient status, hospital management, cost, and national health policy.^[6,16,30]^ Colonization by VRE or CRE could be asymptomatic; however, these colonizing organisms last for a very long period and act as a reservoir for transmission to other patients.^[4,5,8]^ Certain VRE- or CRE-colonized patients are at risk of infection, especially immunocompromised patients and those with long periods of antibiotic use.^[8,17]^ Bloodstream infections of these bacteria are associated with an increased number of invasive procedures, additional antimicrobial therapy, and significantly elevated mortality.^[12,16,32]^ Furthermore, VRE- and CRE-colonized patients should be isolated according to the current infection control policy and cannot receive active rehabilitation therapy in a rehabilitation room. Only simple bedside exercise in an isolation room is allowed, and these patients are suffered and deprived of the chance for functional improvement. Thus, patients could lose an important period for functional recovery after disease. Ultimately, colonization of VRE or CRE results in an extended length of hospital stay and increases the total hospital costs.

### 4.4 Factors related to successful decolonization

This trial is the first study to evaluate the success rate of decolonization protocols associated with functional status. Many previous studies have presented the risk factors related to MDRO colonization or decolonization. However, functional factors regarding ambulation, swallowing, nutrition, or voiding factors were not evaluated. Our results showed that higher FAC scores, higher grip power and higher BMI were significantly associated with success rates in patients. These ambulatory function, grip power, and BMI factors were used for standard diagnostic scales for sarcopenia. Our results revealed that patients with a good functional status had an increased likelihood of successful decolonization. In other words, if patients could not receive active rehabilitation within the golden time because of MDRO isolation, a more functional decrease would be accelerated, creating a vicious cycle. Thus, we suggest that active rehabilitation therapy for functional improvement in isolated patients should be considered.

Interestingly, a shorter MDRO isolation period without a trial was significantly associated with the success rate of the decolonization protocol. Thus, as the Centers for Disease and Control and Preventions of many countries have emphasized, active surveillance for early identification of carriers and early decolonization protocol initiation are crucial.^[17,19,20]^

### 4.5 Study limitations

This study is a preliminary investigation with a small number of patients. Because our protocol was comprehensive and multisystem, the cooperation of patients or caregivers was essential. Thus, identifying a control group was difficult, and the lack of a control group could limit the validity of the results. We could not classify and confirm the cultured VRE and CRE colonies by PCR for DNA sequencing. Thus, the difference in the effectiveness of our decolonization protocol ranked according to the cultured colonies was not established.

## 5 Conclusion

This study demonstrated successful decolonization of VRE and CRE organisms via a comprehensive, multisystem, mechanical decolonization protocol. Most importantly, our protocol did not use antibiotics and showed functional factors related to successful decolonization. Through our results, it is critical to conduct an active decolonization trial as early as possible in patients with VRE or CRE colonization. These strategies could also be potential alternative solutions for other MDROs that colonize the GI tract. Furthermore, this protocol can be used as 1 of the basic treatment options for MDROs infection or colonization, regardless of whether it requires antibiotic treatment at a later stage. Our simple, safe, and easy-to-apply decolonization protocol shows promise and may significantly impact MDRO decolonization strategies in clinical settings.

## Author contributions

Sook Joung Lee and Eunseok Choi: Study design, Data collection, Manuscript preparation,

S. Lee, S. Jang, H.Y. Jeong and Y. Joo: Literature search, Data collection

J. Yi and Y. S. Lee: Study design, Analysis of data, Review of the manuscript

**Conceptualization:** Eunseok Choi, Sook Joung Lee, Jinseok Yi.

**Data curation:** Eunseok Choi, Sangjee Lee, So-youn Chang, Ho Young Jeong, Yunwoo Joo.

**Formal analysis:** Sangjee Lee, Jinseok Yi, Yeon Soo Lee.

**Investigation:** Sook Joung Lee, Yeon Soo Lee, So-youn Chang.

**Project administration:** So-youn Chang, Ho Young Jeong, Yunwoo Joo.

**Resources:** Sangjee Lee.

**Supervision:** Eunseok Choi.

**Validation:** Jinseok Yi, Yeon Soo Lee.

**Writing – original draft:** Eunseok Choi, Sook Joung Lee.

**Writing – review & editing:** Eunseok Choi, Sook Joung Lee.
